# Modeling pediatric antibiotic use in an area of declining malaria prevalence

**DOI:** 10.1038/s41598-024-67492-x

**Published:** 2024-07-16

**Authors:** Lydia Helen Rautman, Daniel Eibach, Felix Osei Boateng, Charity Wiafe Akenten, Henry Hanson, Oumou Maiga-Ascofaré, Jürgen May, Ralf Krumkamp

**Affiliations:** 1 Department of Infectious Disease Epidemiology, Bernhard Nocht Institute for Tropical Medicine, Bernhard-Nocht-Str 74, 20359 Hamburg, Germany; 2 University Medical Center Hamburg-Eppendorf, Hamburg, Germany; 3 German Center for Infection Research, Hamburg-Borstel-Lübeck-Riems, Hamburg, Germany; 4 Kumasi Centre for Collaborative Research in Tropical Medicine, Kumasi, Ghana

**Keywords:** Probability model, Decision tree, Antibiotic reduction, Malaria endemicity, Malaria, Paediatric research, Epidemiology, Antimicrobial therapy

## Abstract

In malaria-endemic areas of Sub-Saharan Africa, overlap of clinical symptoms between malarial and non-malarial febrile illnesses can lead to empiric use of antibiotics among children. Our study aimed to illustrate the potential impact of decreasing malaria prevalence from malaria control efforts on antibiotic use. We constructed a probabilistic decision tree model representing antibiotic prescription in febrile children < 5 years. This model was used to predict change in absolute antibiotic use compared to baseline under levels of decreasing malaria prevalence. Model parameters were based on data from a hospital study in Ghana and validated via literature review. The baseline prevalence of malaria diagnoses was 52% among all hospitalized children. For our main results, we reported outcomes for a scenario representing a 50% decrease in malaria prevalence. Compared to baseline, absolute antibiotic prescription decreased from a baseline of 639 doses (95% CI 574–694) to 575 (95% CI 502–638). This reflected a 10% (95% CI 7%–13%) decrease in absolute antibiotic use. Our findings demonstrate that effective malaria control can reduce pediatric antibiotic use. However, until substantial progress is made in developing accurate diagnostics for non-malarial febrile illnesses, further reductions in antibiotic use will remain a challenge.

## Introduction

Through great efforts over the last decades, quality and availability of medications and healthcare in Sub-Saharan Africa have improved, making it easier to seek care for and treat childhood febrile diseases. Access to diagnostics has also increased, most notably with the widespread distribution of the malaria rapid test, and these successes have led to global decreases in malaria incidence^1^.

In areas where malaria is endemic, the significant clinical overlap of non-specific symptoms that accompany malaria and non-malarial febrile illnesses (NMFIs) can make it challenging to differentiate between the two. Especially in severe cases where delays can result in long-term sequelae or mortality, treatment is often initiated before laboratory results are available and children with malaria can be treated, sometimes empirically, with antibiotics^2–4^. Antibiotics are not necessary for the treatment of uncomplicated malaria and are only recommended where a bacterial co-infection has been confirmed or a severe bacterial infection such as sepsis is suspected^5,6^. However, the World Health Organization (WHO) additionally recommends the use of antibiotics for treating suspected severe malaria^6^, adding complexity to the goal of reducing antibiotic over-treatment. Misuse of antibiotic medications can result in the development of resistance, as well as drug toxicity and financial burden on the individual and the health care system^7,8^.

Characterizations of the relationship between malaria prevalence and antibiotic use at a population level have emerged in the literature, with one study in Uganda describing a decrease in antibiotic treatments following intensive malaria control efforts^9^. Drug prescribing practices will be impacted by global decreases in malaria transmission with continued public health efforts. Massive reductions in malaria incidence since 2000 can be attributed to the development and application of vector control and pharmaceutical interventions. New strategies, such as malaria vaccines, present opportunities for the prevention and control of malaria in an effective and easily implementable way not previously available. In mathematical models, implementation of mass vaccination strategies alongside chemoprevention drastically reduces prevalence and can, in some cases, interrupt transmission^10^. However, to what extent this will impact the overall treatment of childhood fever is unclear. Our study aimed to illustrate the effects of changing malaria endemicity on prescribing practices, specifically on antibiotic prescription.

## Methods

### Mathematical model structure

**Figure 1:**
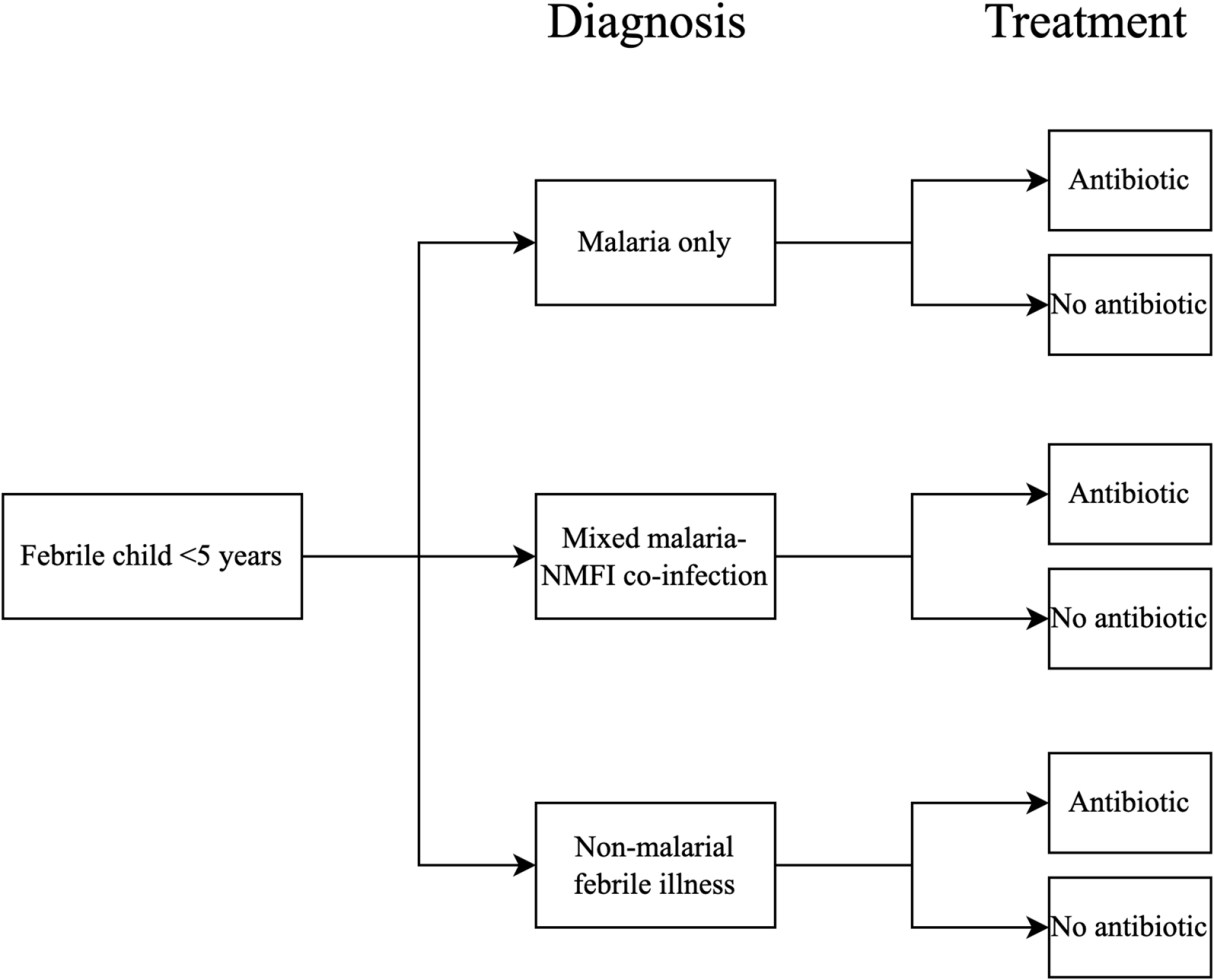
Decision tree model structure. NMFI non-malarial febrile illness.

A probabilistic decision tree model was developed to estimate the effect of decreasing malaria prevalence on antibiotic use among febrile children aged < 5 years. The model was constructed to represent a hospital in a low-income country situated in a malaria-endemic area and mirrored current diagnostic practices in the study hospital. Malaria rapid diagnostic tests (RDTs) were performed for all children with fever, except during occasional shortages, and microscopy was available to confirm positive malaria RDTs. Full blood count was done, and urine dip sticks and rapid tests were used for relevant infections (e.g., HIV, typhoid, hepatitis B). Bacterial cultures were available but were rarely performed. Diagnoses for NMFIs were frequently clinical. The structure of our treatment decision tree (Fig. 1) follows flows based on clinical diagnosis and treatment decisions. Probability parameters illustrate the distribution of the underlying diagnosis and treatment in a population of hospitalized febrile children. Each simulation run represented a population of 1,000 admitted febrile individuals. Scenarios were constructed to show the effect of different reductions in local malaria prevalence. A baseline scenario (A) was parameterized, which represents the actual diagnosis and treatment prevalence among hospitalized children < 5 years in a rural area in Ghana^11^. Further reductions (scenarios) were simulated and compared to the baseline Scenario A. This model was constructed based on categories needed to answer the study question: diagnostic categories consisted of a malaria-only diagnosis, a mixed malaria-NMFI diagnosis, and an NMFI-only diagnosis. We did not differentiate between uncomplicated malaria and severe malaria cases because the frequencies are interdependent and can be displayed within a single group. For treatment, we distinguished only between children who received an antibiotic and those who did not.

The scenarios simulated reductions of malaria diagnosis prevalence by increments of 10%: for example, the difference between Scenarios A and B was calculated as if 10% of the malaria infections in Scenario A had been prevented. This meant that 10% of children with a malaria-only diagnosis were removed from this compartment. Accordingly, 10% of children with a mixed infection were no longer infected with malaria and moved into the NMFI-only diagnosis compartment. We assumed that co-infections were independent of malaria infections. Scenario C modelled a 20% decrease, Scenario D a 30% decrease, and so on, through Scenario J, which represented the prevention of 90% of malaria cases compared to baseline. The increments used to model different levels of reduction were arbitrary but facilitated a more comprehensive understanding of the relationship between malaria prevalence and antibiotic use across a range of hypothetical values.

For parameterization of the model, we employed a Monte Carlo (MC) simulation and generated 10 000 random parameter samples from a Beta distribution. Specific equations for the model structure are given in the supplement. MC simulations were run for each prevalence scenario. Model results were summarized with a point estimate (median) and 95% confidence interval (0.025 and 0.975 percentile) from simulation outputs. Diagnosis prevalence, total febrile population, relative and absolute antibiotic use, and change in absolute antibiotic use compared to baseline were also reported for each scenario.

### Model parameterization

Parameter estimates relating to the underlying disease distribution were taken from a study on hospitalized febrile children conducted at Agogo Presbyterian Hospital (APH), Ghana between November 2013–December 2015. The study protocol and specific methods are described elsewhere^11^.

APH is a 350-bed district hospital in the Asante Akim North District in Ghana, an area in which malaria is hyperendemic. Hospital discharge diagnosis and treatment data for febrile children < 5 years admitted to the pediatric ward with non-missing treatment data were used to derive baseline parameter estimates. We validated these with parameter estimates found via literature review. MEDLINE database was searched via PubMed from years 2003–2023 using the search terms “(malaria) AND (antibiotic) AND (Africa)” and filtering for full-text results only. We reviewed all publications and excluded reviews, mathematical studies, commentaries, case reports, and mortality reports, as well as study populations not including children, those smaller than 500 individuals, and those focusing on individuals living with HIV. Studies with a study population that was not exclusively children were included if the proportion of the study population under 15 years was greater than 30%.

To address the uncertainty of parameter estimates in our model, we performed a sensitivity analysis on diagnosis and antibiotic treatment parameters to identify the parameters most integral to the model. Because the diagnosis categories were mutually exclusive, diagnostic prevalence values were inter-dependent and effects could be observed in the prevalence of all parameters by varying the prevalence of only one; in our sensitivity analysis, only the prevalence of malaria-only diagnoses was varied.

### Ethics approval and consent to participate

The study in which data were collected for this model study and the informed consent procedures were approved by the Committee on Human Research, Publications and Ethics, School of Medical Science, Kwame Nkrumah University of Science and Technology, Kumasi, Ghana (CHRPE/AP/427/13) and the Ethics Committee of the Ärztekammer Hamburg, Germany (PV4592). Prior to enrollment, written informed consent was obtained from the parents or guardians of that child.

## Results

### Model parameters

Data from 657 children were used to estimate parameters. The baseline prevalence of malaria diagnoses was 52%, which broke down to 38% malaria-only diagnoses and 14% mixed diagnoses; the remaining 48% were NMFI-only diagnoses. Antibiotic prescription prevalence was overall 64%, with 38% among malaria-only diagnoses, 71% among mixed diagnoses, and 82% among NMFI-only diagnoses.

For the literature review, after removal of duplicates, our search yielded a total of 579 results. Four hundred and thirty-seven papers were eliminated based on the title; 45 and 80 were excluded after reading the summary and the full text, respectively. The remaining 17 studies were analyzed in-depth and five were excluded due to lack of relevant parameters. Table 1 illustrates the validation of parameter baseline values among the twelve studies gathered in the literature review. Simple averages of parameters taken from studies obtained via literature review are presented for comparison with parameter estimates from our own study: literature estimates of antibiotic prevalence among malaria diagnoses were consistent with those from our study (both 64%) and antibiotic prevalence among NMFI diagnoses was slightly lower in the literature compared to our study (75% vs. 82%). Diagnostic prevalence estimates were context-specific, although the malaria prevalence in another study in Ghana was comparable to our prevalence (56% vs. 52%). None of the selected studies found via literature review captured the prevalence of antibiotic use among mixed diagnoses.

**Table 1:** Literature review on parameter baseline values for validation of study estimates (by most recent).

Data source	Country	Study population	Study size	Malaria diagnostic method	Malaria diagnosis prevalence	NMFI diagnosis prevalence	Antibiotic prescription prevalence	AB prescription among NMFI diagnoses	AB prescription among malaria diagnoses
Kapisi et al.^12^	Uganda	Febrile outpatients ≥ 1 year	981^1 2^	RDT	48% (473/981)	52% (508/981)	56% (546/981)	74% (376/508)	36% (170/473)
Kourouma et al.^3^	Guinea	Febrile in- and outpatients, all ages^3^	23,583	RDT or microscopy	61% (14,465/23,583)	39% (9118/23,583)	63% (14,834/23,583)	81% (7402/9118)	51% (7432/14,465)
Hooft et al.^2^	Kenya	Febrile in- and outpatients, 1–17 years	5737	RDT	53% (3065/5737)	47% (2672/5737)	68% (3902/5737)	89% (2287/2577)	53% (1615/3065)
Fomba et al.^13^	Mali	Febrile outpatients, < 5 years	1602^1^	RDT or microscopy	36% (573/1602)	64% (1029/1602)	64% (1018/1585)	68% (683/1012)	59% (335/573)
Bonko et al.^14^	Burkina Faso	Febrile in- and outpatients, < 5 years	1097	Microscopy	54% (589/1097)	46% (508/1097)	78% (856/1097)	91% (462/508)	67% (394/589)
Ahiabu et al.^15^	Ghana	All outpatient prescriptions^4^	1600	RDT or microscopy or clinical diagnosis	56% (893/1600)	44% (707/1600)	60% (958/1600)	67% (473/707)	54% (485/893)
Johansson et al.^16^	Malawi	Febrile outpatients, < 5 years	1981	RDT	35% (769/2205)	64% (1406/2205)	65% (1439/2205)	80% (1131/1406)	37% (282/769)
Onchiri et al.^17^	Kenya	Febrile in- and outpatients, 6 months-15 years	1476	RDT or microscopy	29% (428/1476)	71% (1048/1476)	82% (1214/1476)	93% (976/1048)	56% (238/428)
Ndhlovu et al.^18^	Zambia	Outpatients with fever^5^	608^1^	RDT	38% (230/608)	62% (378/608)	52% (316/608)	65% (247/378)	30% (69/230)
Bastiaens et al.^19^	Tanzania	Febrile outpatients, < 10 years	1608^1 6^	RDT	16% (252/1581)	84% (1329/1581)	60% (940/1581)	65% (865/1329)	30% (75/252)
Batwala et al.^20^	Uganda	Febrile outpatients, < 5 years	8552	RDT or microscopy	19% (852/4336)	80% (3485/4336)	68% (2966/4336)	74% (2608/3485)	42% (358/852)
Bisoffi et al.^21^	Burkina Faso	Febrile outpatients, ≥ 6 months	1058	RDT	53% (556/1054)	47% (494/1054)	53% (560/1050)	57% (281/494)	49% (274/556)
Hogan et al.^11^	Ghana	Febrile inpatients < 5 years	657	RDT or microscopy	52% (343/657)	48% (314/657)	64% (419/657)	82% (258/314)	47% (161/343)
Simple average excluding Hogan et al. (2018)					42%	58%	64%	75%	47%
Difference from Hogan et al. (2018)					Context-dependent	Context-dependent	+ 0%	− 7%	+ 0%

*AB* antibiotic, *NMFI* non-malarial febrile illness, *RDT* rapid diagnostic test.

Italics denote the study from which our model parameters were estimated.

^1^N is a subset of patients with RDT results available.

^2^Control arm only included in this analysis.

^3^Proportion children < 15 was 51% (n/N = 11,987/23,583).

^4^Proportion children < 15 was 38% (n/N = 582/1600).

^5^Proportion children < 5 was 50% (n/N = 437/872).

^6^Intervention was a policy change to restricting antimalarial use only to children with a positive RDT; both arms are included in this analysis.

For model inputs, parameter values estimated from study data were sampled from a Beta distribution; details are given in Table 2. Within the model structure, the prevalence of mixed diagnoses (*P*_*Mixed*_) and NMFI diagnoses (*P*_*NMFI*_) were dependent upon the prevalence of malaria diagnoses (*P*_*Malaria only*_). Therefore, only the value for *P*_*Malaria only*_ was sampled from a Beta distribution, while *P*_*Mixed*_ and *P*_*NMFI*_ were calculated based on the sampled value, as given in the equations in the table.

**Table 2:** Input parameter values and distributions.

Parameter	Description	Distribution	Base value	Range	Histogram of parameter estimates following a Beta distribution
${P}_{Malaria only}$	Prevalence of malaria-only diagnoses	Beta(139.0, 226.3)	0.38	0.29, 0.48	
${P}_{Mixed}$	Prevalence of mixed malaria-NMFI diagnoses	$\frac{{P}_{{Mixed}_{Base}}*(1-{P}_{Malaria only})}{1-{P}_{{Malaria only}_{Base}}}$	0.14	0.12, 0.16	
${P}_{NMFI}$	Prevalence of NMFI diagnoses	$\frac{{P}_{{NMFI}_{Base}}*\left({1-P}_{Malaria only}-{P}_{Mixed}\right)}{{1-P}_{{Malaria only}_{Base}}-{P}_{{Mixed}_{Base}}}$	0.48	0.40, 0.55	
${AB}_{Malaria only}$	Prevalence of antibiotic use among malaria-only diagnoses	Beta(139.1, 227.0)	0.38	0.30, 0.47	
${AB}_{Mixed}$	Prevalence of antibiotic use among mixed diagnoses	Beta(64.6, 26.4)	0.71	0.53, 0.86	
${AB}_{NMFI}$	Prevalence of antibiotic use among NMFI diagnoses	Beta(39.3, 8.5)	0.82	0.54, 0.97	

Base parameter values (${P}_{{Malaria only}_{Base}}$*, *${P}_{{Mixed}_{Base}}$*, and *${P}_{{NMFI}_{Base}}$_*)*_ are 0.38, 0.14, and 0.48, respectively. Scenario-specific *P*_*Malaria only*_ is the value sampled from the Beta distribution and *P*_*Mixed*_ is obtained via the calculation given in the table.

*AB* antibiotic (use), *NMFI* non-malarial febrile illness.

### Model results

**Figure 2:**
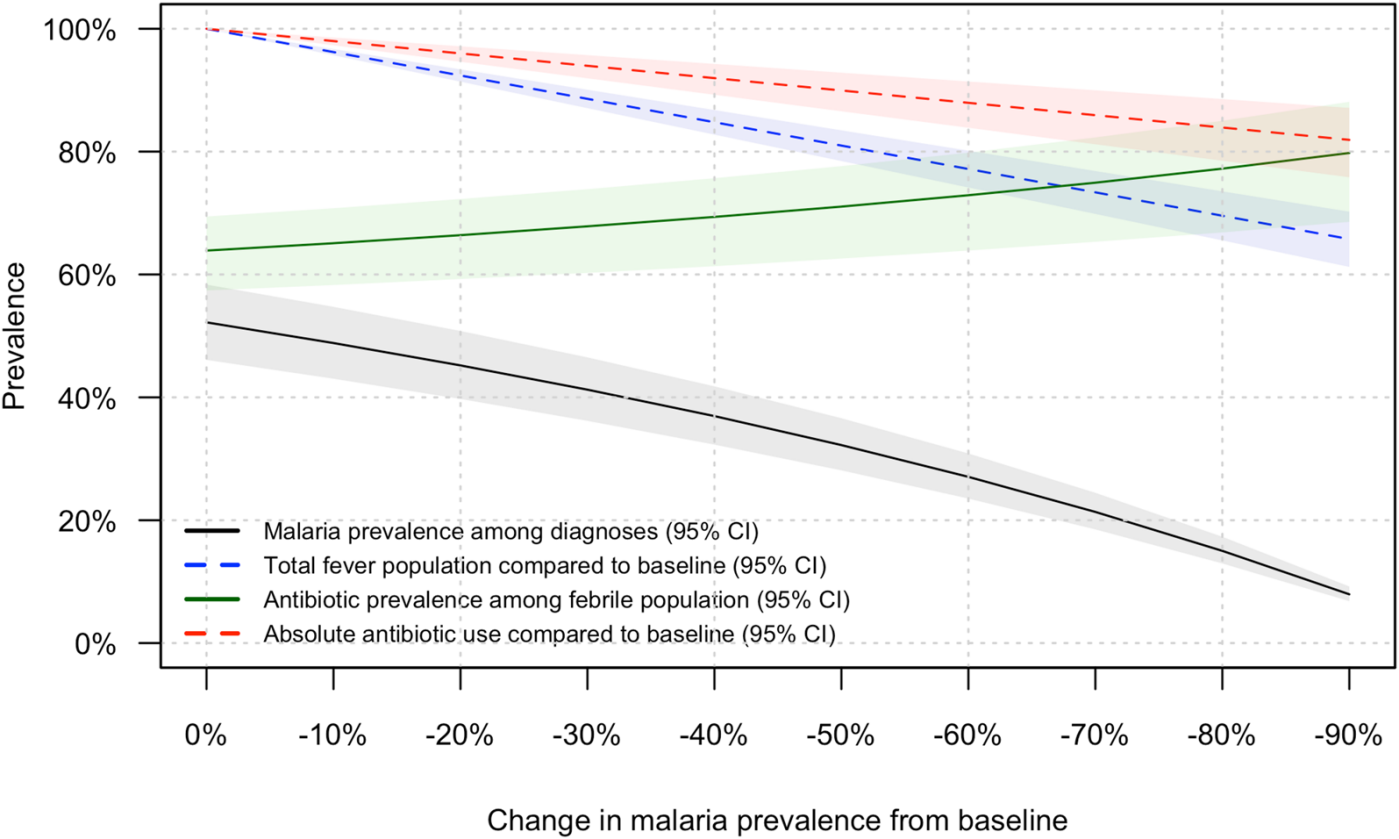
Changes in malaria prevalence and antibiotic use across simulations.

Model results are shown in Table 3. As the prevalence of malaria-only and mixed infections decreased across scenarios, the prevalence of NMFI diagnoses increased proportionally. Across 10 000 simulations, the median number of febrile children in the model decreased 19% (95% CI 17–22%) from 1000 in Scenario A to 810 (95% CI 785–835) in Scenario F, which represented a reduction of malaria prevalence by 50%. The median absolute number of antibiotic prescriptions in Scenario A was 639 (95% CI 574–694), compared with 575 (95% CI 502–638) in Scenario F. This corresponded to a 10% (95% CI 7–13%) decrease in absolute antibiotic prescriptions when malaria prevalence was reduced by 50% (Fig. 2). Because of the reduction in number of febrile individuals, prevalence of antibiotic prescription increased from 64% (95% CI 57–69%) in Scenario A to 71% (95% CI 63–78%) in Scenario F.

**Table 3:** Simulation results of scenarios with changing malaria prevalence.

Scenario	Percent decrease in malaria prevalence from baseline	Diagnostic prevalence			Absolute simulation outcomes		Relative simulation outcomes	
		Malaria only	Mixed malaria-NMFI infection	NMFI only	Absolute antibiotics prescribed	Total febrile population	Percent antibiotics prescribed among total febrile population	Percent change in absolute antibiotic prescription compared to baseline
A	0	38.0% (33.1–43.1%)	14.2% (13.0–15.3%)	47.8% (43.9–51.6%)	639 (574–694)	1000 (1000–1000)	63.9% (57.4–69.4%)	0.0% (0.0–0.0%)
B	10	35.6% (30.8–40.5%)	13.2% (12.2–14.2%)	51.2% (47.3–55.0%)	626 (560–682)	962 (957–967)	65.1% (58.3–70.8%)	− 2.0% (− 2.7 to − 1.4%)
C	20	32.9% (28.3–37.7%)	12.3% (11.4–13.1%)	54.8% (50.9–58.6%)	614 (545–671)	924 (914–934)	66.4% (59.3–72.3%)	− 4.0% (− 5.4 to − 2.9%)
D	30	30.1% (25.7–34.6%)	11.2% (10.5–11.9%)	58.8% (54.9–62.4%)	601 (531–660)	886 (871–901)	67.8% (60.3–73.9%)	− 6.0% (− 8.1 to − 4.3%)
E	40	26.9% (22.9–31.2%)	10.0% (9.4–10.6%)	63.1% (59.4–66.5%)	588 (517–649)	848 (828–868)	69.4% (61.4–75.7%)	− 8.0% (− C10.7 to − 5.7%)
F	50	23.5% (19.8–27.4%)	8.7% (8.3–9.2%)	67.8% (64.3–71.0%)	575 (502–638)	810 (785–835)	71.0% (62.6–77.6%)	− 10.1% (− 13.4 to − 7.2%)
G	60	19.7% (16.5–23.2%)	7.3% (7.0–7.6%)	72.9% (69.8–75.8%)	562 (488–626)	772 (742–801)	72.9% (63.9–79.8%)	− 12.1% (− 16.1 to − 8.6%)
H	70	15.6% (12.9–18.5%)	5.8% (5.6–6.0%)	78.6% (75.9–81.1%)	549 (473–615)	734 (699–768)	74.9% (65.3–82.3%)	− 14.1% (− 18.8 to − 10.0%)
I	80	10.9% (9.0–13.1%)	4.1% (4.0–4.2%)	85.0% (82.9–86.8%)	537 (458–604)	696 (655–735)	77.2% (66.8–85.0%)	− 16.1% (− 21.5 to − 11.5%)
J	90	5.8% (4.7–7.0%)	2.1% (2.1–2.2%)	92.1% (90.8–93.1%)	524 (443–594)	658 (612–702)	79.8% (68.6–88.1%)	− 18.1% (− 24.2 to − 12.9%)

Values given are the median and 95% confidence interval of simulation outputs.

*NMFI* non-malarial febrile illness.

### Sensitivity analyses

**Figure 3:**
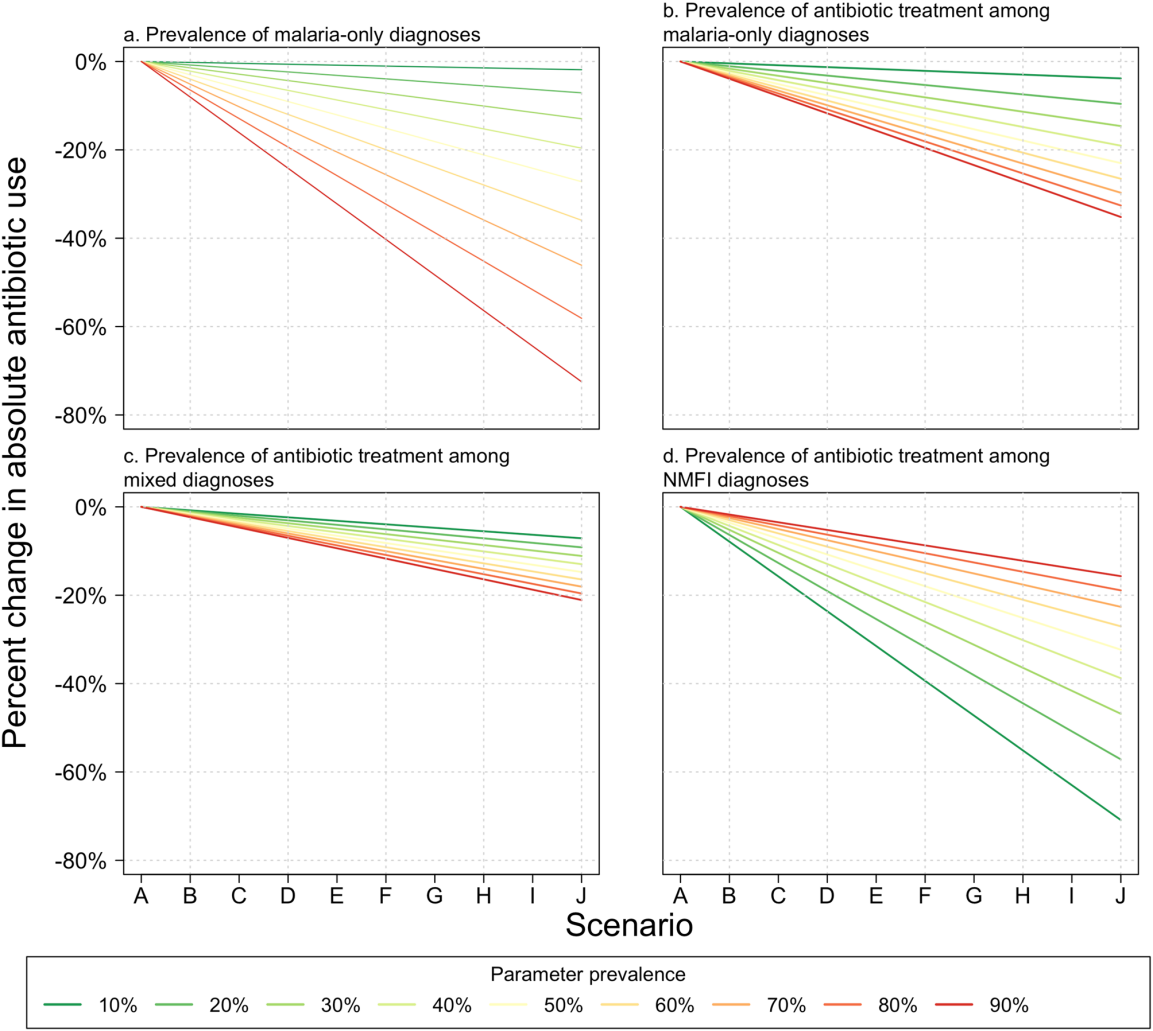
Sensitivity analysis of diagnostic and treatment parameters. Figures show the percent change in absolute antibiotic use under varying prevalence of: (**a**) malaria-only diagnoses, (**b**) antibiotic treatment among malaria diagnoses, (**c**) antibiotic treatment among mixed diagnoses, and (**d**) antibiotic treatment among non-malarial febrile illness diagnoses. *FI* non-malarial febrile illness.

Our sensitivity analyses illustrated the individual impact of the diagnostic and antibiotic use parameters on absolute antibiotic use. For all simulated values of disease distribution and antibiotic treatment, absolute antibiotic use decreased across scenarios (Fig. 3). Absolute antibiotic use was most sensitive to changes in the distribution of diagnoses and in the use of antibiotics to treat NMFIs. Decreases in absolute antibiotic use across scenarios were greater when the starting malaria prevalence was higher. The Supplementary Table details the results of the sensitivity analysis in a table, with the outcome being the percent change in absolute antibiotic use between Scenarios A and J for that parameter value.

## Discussion

### Advancements in malaria combat

Between 2000–2022, malaria incidence in the WHO Africa Region declined 40%^1^. With diagnostics, medications, and vaccines much more advanced compared to those available two decades ago, further reductions of malaria cases – for example by one-half, as shown in our model – are certainly within reach. The WHO Technical Strategy for Malaria details the pillars by which malaria incidence can be further reduced: in addition to the improvement of surveillance tactics, vector control strategies, combating the development of resistances, and malaria vaccine rollout are critical components in the pursuit of this goal^22^.

Insecticide-treated nets and indoor residual spraying are the two primary strategies for vector control but are frequently combined with other vector control or pharmaceutical strategies to further reduce malaria incidence. Recent resistances to antimalarial therapies have been documented with reports of increased treatment failure rates and artemisinin resistance^1^, but active measures including optimizing and regulating antimalarial medications, developing novel antimalarial drugs, and afore-mentioned vector control are effective in limiting the spread of resistance^23^. Finally, the RTS,S/AS01 and R21/Matrix-M vaccines represent major breakthroughs in malaria vaccine development as the first vaccines to be approved by the WHO for use against malaria and have 12-month vaccine efficacies of 56% and 68%, respectively^24,25^. Large-scale rollouts have begun in early 2024 and soon, malaria vaccines will be available to millions of children living in areas of moderate and high malaria transmission.

### Synthesis of model findings

Changes in the prevalence, detection, or treatment of one disease can affect the broader disease and treatment landscape. Intuitively, antimalarial use will decrease with declining malaria prevalence, but because of the clinical overlap of malaria and other childhood febrile illnesses it is crucial to also consider the potential effect on antibiotic use. Findings from our mathematical model suggest that as malaria prevalence decreases, the relative prevalence of antibiotic use among fever cases will rise, yet absolute antibiotic use will decrease due to the reduction of total fever cases. These changes in prescription practices could mean the avoidance of hundreds of thousands of antibiotic courses in Sub-Saharan Africa, where the pediatric population is expected to reach 1 billion by 2055^26^. This underscores the dynamic impact of malaria elimination efforts in the alteration of patterns of drug use.

In parts of Sub-Saharan Africa, inappropriate antimicrobial use and over prescription is becoming a pressing issue, especially among children^2–4,27^. An antibiotic that is incorrectly dosed or inappropriately used can result in drug toxicity, delayed treatment of the actual infection, and, most concerningly, can contribute to the development of antibiotic resistances^7^. Our model did not aim to evaluate the justification of treatment choices; in some cases, empiric antibiotic treatment might be necessary to avoid treatment delays of a potentially fast-progressing and serious condition, such as sepsis or meningitis. Instead, our goal was to provide an objective depiction of how reductions in malaria transmission could impact overall drug use. The epidemiologic landscape will continue to change with declining malaria transmission, bringing new challenges to accurate and timely diagnosis. Malaria RDTs are currently used to differentiate between malaria and NMFIs, resulting in the widespread treatment of malaria RDT-negative patients with antibiotics^14,28^. A lack of diagnostic tools to further differentiate between bacterial and viral NMFIs presents a challenge in targeting antibiotic treatment and with the further reduction of malaria transmission, this problem will become more pronounced. Improving decision algorithms, strengthening laboratory capacities, and developing more specific tools such as rapid bacteriological diagnostics and biomarkers will enhance targeted treatment for antibiotic-requiring infections^29–31^ and could save lives through improved case detection.

### Considerations and limitations

As malaria cases become less frequent, the age spectrum will shift to older children, and interventions toward young children may need to be adjusted^32^. Diagnostic accuracy, especially of clinical diagnoses, might also decline. With a constantly changing disease landscape, it could be advantageous for hospitals to use epidemiologic surveillance data to regularly assess the local disease prevalence. Data-driven approaches can be used to enhance clinical diagnoses by providing statistical insights, particularly in settings where diagnostic tools are lacking.

Our parameters are an estimation of the true value of diagnosis and treatment prevalence, so corresponding 95% confidence intervals provide more cautious estimates of the values and become wider with decreasing malaria prevalence. Our parameters were informed by data taken from a study conducted at APH, therefore the simulated distribution of disease and diagnoses might not be directly applicable to other settings, as practices are likely to differ between institutions and cultures. However, our literature review validated our parameter estimates; additionally, the broader discussion surrounding this topic remains relevant for settings where malaria prevalence is decreasing.

While our model illuminates one effect of decreasing malaria transmission, other factors might help to provide a more comprehensive illustration of the effects on antibiotic prescription. Malaria-dependent co-infections, such as invasive Salmonella, were not considered in the model structure but exist nonetheless and could be included in future models. As malaria-dependent co-infections would decline at the same rate as malaria prevalence (without a proportional increase in NMFI diagnoses, as seen with independent coinfections), our current model underestimates changes in antibiotic prescription and inclusion of dependent co-infections would result in even larger decreases in antibiotic use.

Modelled scenarios of specific interventions can be informative but are highly context specific. Efficacy estimation of combined malaria control approaches is especially challenging because the effect is not simply additive but more complex^10,33^. Thus, we chose to model arbitrary levels of decreased transmission to maximize the applicability of our findings. As additional efficacy studies are conducted for the vaccines and their combination with other malaria control strategies, increased confidence in these estimates will enhance our ability to apply these in interpreting model results.

We examined our model in a hospital setting because of the frequency of antibiotic use for severe disease and data availability, although still limited. Most of the studies in our literature review focused on outpatients, demonstrating the similarity in prescribing practices between outpatients and the inpatients in our study, although further research is needed to more thoroughly assess this. It is also relevant to consider how our model would perform in the informal sector, for instance with data from pharmacies or drug sellers. Antibiotics are frequently bought over the counter, but effects of this are difficult to capture due to lack of data, particularly to the underlying diagnosis, as no professional care is sought. However, a different dynamic is to be expected here, as antibiotic purchasing is dependent upon the market and the earning potential of the local pharmacies^34^. Better data to this topic is important, not only for research but also for policy makers, to better understand the use of antibiotics and to implement targeted interventions for the improvement of self-treatment practices.

## Conclusion

Reductions in malaria transmission result in decreases in absolute antibiotic use. Findings from our model illustrate this relationship and underscore its importance in predicting the trajectory of prescription drug use. Continued implementation of malaria interventions will give way to reductions in malaria prevalence and will additionally reduce antibiotic use.

### Supplementary Information

Supplementary Information.

## Data Availability

De-identified data were published with a separate manuscript and are available for download from the open-access data repository *Zenodo* at 10.5281/zenodo.8220138. Model code is available at 10.5281/zenodo.11656911.

## Abbreviations

AB: Antibiotic
APH: Agogo Presbyterian Hospital
CI: Confidence interval
HIV: Human immunodeficiency virus
MC: Monte Carlo
RDT: Rapid diagnostic test
NMFI: Non-malarial febrile illness
WHO: World Health Organization
