# Supplementary material for: Modeling pediatric antibiotic use in an area of declining malaria prevalence
Source: Sci Rep. 2024 Jul 16;14:16431. doi: 10.1038/s41598-024-67492-x (PMC11252319; doi:10.1038/s41598-024-67492-x)
Supplement: Supplementary file 1 — Supplementary Information. [file 41598_2024_67492_MOESM1_ESM.docx]

**Supplement to methods**

We employed Monte Carlo simulations and generated 10 000 random parameter samples from a Beta distribution with the probability density function: $f\left( x;\alpha, \beta\right)= \frac{x^{\alpha-1}{(1-x)}^{\beta-1}}{B(\alpha,\beta)}$ with bounds [0,1] and shape parameters $\alpha$ and $\beta$ corresponding to the mean of the probability density function, equal to the base input value, and solved from mean $\mu= \frac{\alpha}{\alpha+\beta}$ and variance $\sigma^{2}= \frac{\alpha\beta}{\left( \alpha+\beta\right)^{2}(\alpha+\beta+1)}$ so that $\alpha=\left( \frac{1-\mu}{\sigma^{2}}-\frac{1}{\mu} \right)\mu^{2}$ and $\beta= \alpha(\frac{1}{\mu}-1)$. Point estimates (median) and 95% confidence intervals were reported from aggregated simulation outputs.

The outcome of interest was the percent change in absolute antibiotic use from baseline for each scenario. Scenario-specific total absolute antibiotic use (${AB}_{{Total}_{S}}$) was comprised of contributing antibiotic use prevalence for each diagnosis (${CAB}_{Diagnosis}$), characterized as a function of the diagnostic prevalence ($P_{Diagnosis}$) and diagnosis-specific antibiotic use (${AB}_{Diagnosis}$):

$${AB}_{{Total}_{s}}=\left( {CAB}_{{Malaria only}_{S}}+{CAB}_{{Mixed}_{S}}+{CAB}_{{NMFI}_{S}} \right)$$

*where*

$${CAB}_{{Malaria only}_{S}}={AB}_{{Malaria only}_{A}}*\frac{P_{{Malaria only}_{A}}*(1-r_{s})}{1-{(P}_{{Malaria only}_{A}}*r_{s})}$$

$${CAB}_{{Mixed}_{S}}={AB}_{{Mixed}_{A}}*\frac{P_{{Mixed}_{A}}*(1-r_{s})}{1-{(P}_{{Malaria only}_{A}}*r_{s})}$$

$${CAB}_{{NMFI}_{S}}={AB}_{{NMFI}_{A}}*\frac{P_{{NMFI}_{A}}+\left( P_{{Mixed}_{A}}*r_{s} \right)}{1-{(P}_{{Malaria only}_{A}}*r_{s})};$$

*for which*

$$P_{{Malaria only}_{A}}\sim Beta\left( \alpha,\beta\right)$$

$${AB}_{Diagnosis} \sim Beta(\alpha,\beta)$$

and $r_{s}$is the scenario-specific reduction coefficient (0.1 for Scenario B to simulate a 10% decrease, 0.2 for Scenario C, and so on). Thus, the percent change in absolute antibiotic use from baseline for each scenario can be calculated as follows:

$${\Delta AB}_{{Total}_{A\to s}}= \frac{{CAB}_{{Malaria only}_{S}}+{CAB}_{{Mixed}_{S}}+{CAB}_{{NMFI}_{S}}}{{CAB}_{{Malaria only}_{A}}+{CAB}_{{Mixed}_{A}}+{CAB}_{{NMFI}_{A}}}*100$$

$AB$ (antibiotic use) parameters represent the likelihood of antibiotic prescription among patients with a specific diagnosis and are multiplied by the respective diagnostic prevalence to obtain the $CAB$ (contributing antibiotic use) parameter, which represents the proportion of the total population with the specified diagnosis receiving antibiotics. The denominator $1-{(P}_{{Malaria only}_{A}}*r_{s})$, as seen in each CAB equation, adjusts for fluctuations in the febrile population. In the model this is calculated as $1,000-{1,000*(P}_{{Malaria only}_{A}}*r_{s})$ to represent changes in a simulated febrile population of 1,000 individuals. The sum of $CAB$ parts for all possible diagnoses therefore equals the total absolute antibiotic use for a scenario.

Supplementary Table. Sensitivity analysis results for diagnostic and treatment parameters.

|  |  | Diagnosis |  | Antibiotic treatment | | |
| --- | --- | --- | --- | --- | --- | --- |
| Parameter value |  | Malaria |  | Malaria | Mixed infection | NMFI |
| 10% |  | -1.9% |  | -3.8% | -7.1% | -70.9% |
| 20% |  | -7.1% |  | -9.6% | -9.2% | -57.2% |
| 30% |  | -13.0% |  | -14.6% | -11.1% | -46.8% |
| 40% |  | -19.6% |  | -19.1% | -13.0% | -38.8% |
| 50% |  | -27.2% |  | -23.0% | -14.7% | -32.3% |
| 60% |  | -35.9% |  | -26.5% | -16.4% | -27.0% |
| 70% |  | -46.1% |  | -29.7% | -18.1% | -22.6% |
| 80% |  | -58.1% |  | -32.6% | -19.6% | -18.9% |
| 90% |  | -72.5% |  | -35.2% | -21.1% | -15.7% |

Shown is the percent change in absolute antibiotic use from Scenario A to Scenario J for each parameter value (the slope of the lines from Figure 3).

Abbreviation: NMFI, non-malarial febrile illness
